# Squibbs’ Epidermis

**Published:** 1886-01-20

**Authors:** 


SQUIBBS' EPIDERMIS.—This little journal has been discontinued. Dr. Squibbs is arranging to travel. On his return it will probably be resumed with renewed interest.

---

DIED, on the 1st of December last, Dr. J. A. Reed, of Anderson Court House, S. C. He was an esteemed physician and worthy citizen.

Dr. Henry Head, formerly of LaGrange, Ga., died at Birmingham, Ala., recently, of angina pectoris.

